# Supplementary material for: Toxocara species environmental contamination of public spaces in New York City
Source: PLoS Negl Trop Dis. 2020 May 5;14(5):e0008249. doi: 10.1371/journal.pntd.0008249 (PMC7228133; doi:10.1371/journal.pntd.0008249)
Supplement: Attachment — Submitted filename: Toxocara reviewers comments.docx [file pntd.0008249.s001.docx]

Dear Dr Mejia,

Thank you very much for submitting your manuscript "Toxocara species environmental contamination in public spaces in New York City" (#PNTD-D-19-01601) for review by PLOS Neglected Tropical Diseases. Your manuscript was fully evaluated at the editorial level and by independent peer reviewers. The reviewers appreciated the attention to an important problem, but raised some substantial concerns about the manuscript as it currently stands. These issues must be addressed before we would be willing to consider a revised version of your study. We cannot, of course, promise publication at that time.

We therefore ask you to modify the manuscript according to the review recommendations before we can consider your manuscript for acceptance. Your revisions should address the specific points made by each reviewer. 

When you are ready to resubmit, please be prepared to upload the following:

(1) A letter containing a detailed list of your responses to the review comments and a description of the changes you have made in the manuscript.

(2) Two versions of the manuscript: one with either highlights or tracked changes denoting where the text has been changed (uploaded as a "Revised Article with Changes Highlighted" file); the other a clean version (uploaded as the article file).

(3) If available, a striking still image (a new image if one is available or an existing one from within your manuscript). If your manuscript is accepted for publication, this image may be featured on our website. Images should ideally be high resolution, eye-catching, single panel images; where one is available, please use 'add file' at the time of resubmission and select 'striking image' as the file type. 

Please provide a short caption, including credits, uploaded as a separate "Other" file. If your image is from someone other than yourself, please ensure that the artist has read and agreed to the terms and conditions of the Creative Commons Attribution License at http://journals.plos.org/plosntds/s/content-license (NOTE: we cannot publish copyrighted images). 

(4) If applicable, we encourage you to add a list of accession numbers/ID numbers for genes and proteins mentioned in the text (these should be listed as a paragraph at the end of the manuscript). You can supply accession numbers for any database, so long as the database is publicly accessible and stable. Examples include LocusLink and SwissProt.

(5) To enhance the reproducibility of your results, we recommend that you deposit your laboratory protocols in protocols.io, where a protocol can be assigned its own identifier (DOI) such that it can be cited independently in the future. For instructions see http://journals.plos.org/plosntds/s/submission-guidelines#loc-methods

Please note while forming your response, if your article is accepted, you may have the opportunity to make the peer review history publicly available. The record will include editor decision letters (with reviews) and your responses to reviewer comments. If eligible, we will contact you to opt in or out.

While revising your submission, please upload your figure files to the Preflight Analysis and Conversion Engine (PACE) digital diagnostic tool, https://pacev2.apexcovantage.com/ PACE helps ensure that figures meet PLOS requirements. To use PACE, you must first register as a user. Then, login and navigate to the UPLOAD tab, where you will find detailed instructions on how to use the tool. If you encounter any issues or have any questions when using PACE, please email us at figures@plos.org.

We hope to receive your revised manuscript by Jan 11 2020 11:59PM. If you anticipate any delay in its return, we ask that you let us know the expected resubmission date by replying to this email.

To submit a revision, go to https://www.editorialmanager.com/pntd/ and log in as an Author. You will see a menu item call Submission Needing Revision. You will find your submission record there. 

Sincerely,

Celia Holland
Guest Editor
PLOS Neglected Tropical Diseases

Christine Budke
Deputy Editor
PLOS Neglected Tropical Diseases

***********************

Thank you for considering our article. Attached are the replies to the reviewer comments.

We hope the paper is now ready for acceptance.

Rojelio Mejia

Reviewer's Responses to Questions

**Key Review Criteria Required for Acceptance?**


As you describe the new analyses required for acceptance, please consider the following:


**Methods**


-Are the objectives of the study clearly articulated with a clear testable hypothesis stated?

-Is the study design appropriate to address the stated objectives?

-Is the population clearly described and appropriate for the hypothesis being tested?

-Is the sample size sufficient to ensure adequate power to address the hypothesis being tested?

-Were correct statistical analysis used to support conclusions?

-Are there concerns about ethical or regulatory requirements being met?


Reviewer #1: See general comment to the Authors

Reviewer #2: Details must be provided of sampling methodology (e.g. when during the year, etc) and sampling strategy (type of sample [random, convenience, etc]; sampling frame, etc). As far as I can see the sampling was not representative so may be subject to bias – this should be discussed. The authors say it is a ‘geo-surveillance’ study but provide no information on whether the sample is representative or geographic risk. Some degree of precision needs to be provided for prevalence estimates (i.e. 95% CI) and information should be provided for sample size and justification for sample size. Saying the Bronx has a 67% prevalence of positive samples is difficult to interpret without some measure of precision.

Unfortunately, the samples were collected at different part of the year from different locations. Seasonal variability will be considered in future studies. These limitations have been expanded in the manuscript.

Information on park selection was added.

The Geo-surveillance sampling was representative, this was added to the manuscript.

Prevalence estimates were added.

Sample size calculation was added to the methods section.

Viability testing can be done by egg incubation which is probably a better approach than microscope alone. This requires some discussion as to how useful is microscopy alone.

Discussion has been added to address how useful microscopy alone is.

Reviewer #3: (No Response)

--------------------

**Results**


-Does the analysis presented match the analysis plan?

-Are the results clearly and completely presented?

-Are the figures (Tables, Images) of sufficient quality for clarity?


Reviewer #1: See general comment to the Authors

Reviewer #2: A useful addition to this manuscript would be to use the NHANES Toxocara seropostivity data (if available for these NYC areas which the authors refer to in the text) to map geographic risk and associate this with the findings of this environmental survey. This would thus link environmental contamination risk with actual human exposure. Surely risk of positive samples by area could be modelled statistically? Such modelling could control for factors such as socioeconomic level, etc.

A good idea, although collecting this data will take time and delay the publication of this article. The NHANES data base does not correlate to soil sampling time points. This discrepancy could be significant and obstruct our findings. vv This article is part of a series of environmental parasite studies that include rural areas of the USA, Ecuador, and Argentina. The goal will be to prospective studies with recombinant Toxocara ELISA developed in our Houston Labs to current environmental samples.

Table 1 should provide more information showing number of samples and number of locations, proportions of samples positive from each location (parks vs. playgrounds, by microscopy, PCR, or either), whether any sample was positive at each location, etc.

Have updated Table 1.

Figure 3 indicates that qPCR correlates perfectly with egg count. Is this true?

Yes, as stated the calculated egg count was from diluted known amounts. It was meant as an estimate and not an exact correlation.

Figure 5 should show medians rather than mean. One outlier for the Bronx biases the mean.

It is in geometric means which takes into account outliers.

Reviewer #3: (No Response)

--------------------

**Conclusions**


-Are the conclusions supported by the data presented?

-Are the limitations of analysis clearly described?

-Do the authors discuss how these data can be helpful to advance our understanding of the topic under study?

-Is public health relevance addressed?


Reviewer #1: See general comment to the Authors

Reviewer #2: Limitations need to be discussed more extensively (see comments above)

These have been addressed in the manuscript.

Reviewer #3: (No Response)

--------------------

**Editorial and Data Presentation Modifications?**


Use this section for editorial suggestions as well as relatively minor modifications of existing data that would enhance clarity. If the only modifications needed are minor and/or editorial, you may wish to recommend “Minor Revision” or “Accept”. 

Reviewer #1: See general comment to the Authors

Reviewer #2: The introduction could be cut significantly without affecting the message of the manuscript.

We have shorten the intro for clarity.

Reviewer #3: (No Response)

--------------------

**Summary and General Comments**


Use this section to provide overall comments, discuss strengths/weaknesses of the study, novelty, significance, general execution and scholarship. You may also include additional comments for the author, including concerns about dual publication, research ethics, or publication ethics. If requesting major revision, please articulate the new experiments that are needed.

Reviewer #1: The ms PNTD-D-19-01601 describes a study aiming at evaluating the environmental contamination of New York City with the eggs of the zoonotic nematodes Toxocara spp. The study has been carried out properly and it is well described, although I suggest some refinements to the Authors before considering it fully suitable for publication.

General comments

The concept that “infective Toxocara eggs are more predominant in poor socio-economical settings” is redundant throughout the ms and explained and discussed too much. I suggest the authors to give such an explanation of the higher presence in the Bronx rather than in the other boroughs once, in a detailed way, without reiterating it various times. The Introduction should also be reworded accordingly, as the ingestion of infective Toxocara eggs by a child poses relevant sanitary issues regardless the family income or the socioeconomic setting of the borough. A child may put in his/her mouth contaminated fingers in Central Park as also in the Bronx. Than, if some areas are more contaminated than others, this increases the risk but the sanitary importance and consequences are the same. Thus, the Study has the primary aim to evaluate the presence of contamination by Toxocara eggs in NYC and then (secondary objective) to compare its level in the five boroughs (see end of the Author summary).
Examples of repetitions: In the last three lines of the abstract this is reported twice, half of Page 3, first line of Page 4

The manuscript has been shortened and made more concise.

I suggest the Authors to avoid wording like “we” “our”, etc, and rather use an impersonal style.

This has been corrected.

The full name of the parasites (Toxocara canis and Toxocara cati) should be reported only the first time the nematodes are cited, or at the begin of a sentence. All the other times , they should read T. canis or T. cati (abbreviating the genus). Please amend the text accordingly.

This has been corrected.

Specific comments

Author summary
First line: add “respectively” after “cats”
Sentence from “Since” to “York City”: I would say that the need of such research relies on the sanitary impact of Toxocara for both humans and animals, rather than because humans are not definitive hosts of the parasite

Changed as suggested.

Introduction
Half of page 3: ….questions still exist surrounding these enigmatic parasites (and not this enigmatic parasite): Toxocara canis and Toxocara cati are two distinct species.

Removed due to redundancy.
Last three lines: Toxocara should be in italics (as also at the end of Page 9, and in other parts of the text, please check).
Corrected.

Methods and Results

The Methods section should contain details of the areas that have been sampled and the samples that have been examined.

How the areas within the boroughs have been selected

Sample sites were selected at random to maximize coverage of each borough, due to limited access, parts of Brooklyn and Staten Island were not tested.

and the samples have been collected.

This is stated after Sample Collection in Methods:

How many samples per each single area.

This is stated in Sample Collection as “Five samples were collected from each location of the park/playground, each sample was from a different geographic part of the area, attempts were made to sample the entire park equally.”

These details should not be present in the Results section, that instead should include only details on the positive samples.

Details have been moved to Method section.

Delete “(The dog form of Toxocara)” at Page 8

Removed

First two lines of Page 9: cats are source of contamination, not vectors (this term has another meaning in veterinary parasitology). Also, dogs may carry T. cati egg after ingestion of cat faeces, thus the eggs are transported passively in dogs’ intestine and eliminated via the faeces. It is a passive transportation, not an infection as it seems by the sentence. Please reword for clarity.

Reworded for clarity.

This suggests that cats are the predominant reservoir causing contamination in New York City play areas, although dogs can transiently carry *T. cati* from eating cat feces, but are not part of the parasites’ life cycle.

Discussion
Last paragraph: Rodents, birds, rabbit are paratenic hosts, not accidental. It would also be interesting to add at least a brief mention on what other “larvated parasites” were found in the samples.

Changed to:

Other paratenic hosts of this parasite include rodents, birds, or rabbits, which when infected continue to contribute to the lifecycle of the parasite. Finally, other larvated parasites were seen by microscopy but unable to be definitively identified in these samples, posing a potential infectious risk to visiting animals or children.

Reviewer #2: A study potentially of interest to readers of PLoS NTDs providing up-to-date data on environmental contamination with Toxocara spp in NYC. There are significant weaknesses with respect to the information provided on sampling strategy and the data presented.

We have updated both our strategy and limitations to address these weaknesses.

Reviewer #3: The aim of the study by Tyungu and co-workers was to give an overview about the contamination rate with eggs of the zoonotic agent Toxocara spp. in soil of playgrounds in New York City, wherefore soil samples from 91 different playgrounds allocated in all five boroughs of NYC were collected. The prevalence varied between different boroughs. The authors draw the conclusion that the income and therefore socioeconomic conditions might be a valuable explanation for the discrepancies between the boroughs. Furthermore, the authors discriminated between Toxocara canis and T. cati by qPCR, leading to the assumption, that playgrounds are predominately contaminated with eggs of T. cati. 
Overall, the manuscript deals with an interesting topic and provides a comprehensive insight into the contamination rate in New York City with Toxocara eggs. However, I have general concerns about the manuscript in its present form (e.g., data reliability if 100 g vs. 150 g soil was used – see comment below). Methods and results are partially superficial and further information on the study design, e.g. seasonal distribution of sampling, on the statistical analyses and molecular analyses is necessary before it may be recommended for publication. Please consider my comments and recommendations below to improve the manuscript.

Introduction:
The Introduction comprises important aspects for the study, but sometimes a clear structure is missing. Rearranging the chapter with a clear structure will definitively make it easier to follow and will help to enhance the understanding why the study is important. 
We have shorten the paper and made it more concise and to the point.

“Stool” is the term in human medicine, for animals the term “feces” is used in veterinary medicine.

Thank you, we have changed this throughout the paper.

It is now generally accepted that the third-stage larva (L3), not the second stage, is the infective stage (see Brunaska et al. 1995: Toxocara canis: ultrastructural aspects of larval moulting in the maturing eggs. Int J Parasitol. 25:683–90.)
Changed to third stage and added reference.

Regarding reference 15: There are further, more recent, large-scale studies on the association of Toxocara seropositivity and neurocognitive deficits, which could be mentioned here like reference 5, or: Erickson, L. D., Gale, S. D., Berrett, A., Brown, B. L., Hedges, D. W., 2015. Association between toxocariasis and cognitive function in young to middle-aged adults. Folia Parasitol. (Praha) 62:048.

These references have been added.

The general migration route of Toxocara larvae in paratenic hosts should be mentioned as the accumulation in different organs leads to different clinical aspects of the disease.

More details have been added at the end of the first paragraph of the introduction.

I am not sure if the last sentence of the Introduction really reflects the study objectives. Contamination rates of public places give only an overview about a potential risk of infection and cannot automatically be extrapolated to the health disparities between different communities.

Changed to: This is the first geo-surveillance study of Toxocara species in all five boroughs of NYC attempting to address a potential risk of infection within NYC communities by first examining soil from around the city.

Methods:
How was the sampling frequency at the sampling sites? Just once or at different time points during the study period?

At each site, all the samples were collected on the same day. Reworded:

Five samples were collected from each location of the park/playground on the same day, each sample was from a different geographic part of the area, attempts were made to sample the entire park equally.

Analysed samples weighed 100-150 g. This is a huge variation (plus 50% in case of 150 g vs. 100g) and may have influenced the chance of egg recovery and egg recovery rates, possibly leading to biased results. This needs at least to be mentioned in the Discussion. (e.g., were the Bronx samples those with the highest sample weights or were the 6 qPCR positive samples those with the highest weights?). Unfortunately, the samples per playground were combined and weights were taken but not recorded. While not an accurate method of measurement, there was exactly 5 samples per playground and each 15 ml tube was filled to capacity with dirt so the volumes were similar for all playgrounds. This has been added to the limitations.

What was the concentration of Tween20 used for washing? What were the ratios of soil sample and flotation/washing solution?

This has been added to the methods section.

Please give “g” instead of rpm throughout - rpm is not a useful unit because force varies with the radius of the centrifuge. Using g allows other researchers to replicate experiments.
Please use degree Celsius instead of Fahrenheit to be consistent with the rest of the manuscript.

This has been corrected.

Please give the sequence of the reverse primer(s). 
Added.

How was the DNA for the standard curve isolated from Toxocara eggs? With the same procedure used for the samples? And what egg numbers were used to construct the standard curve?

The method and number of eggs were added to the paper.

Statistical analyses: It is unclear what the difference between the two tests (Toxocara eggs by zip code and Toxocara eggs by individual boroughs) is, and why both a Kruskal-Wallis test and an ANOVA was conducted. The ANOVA does not compare prevalence, as egg count data was used (same as in the Kruskal-Wallis test?), please rephrase (prevalence would be presence/absence data). Were post-hoc tests conducted to clarify which boroughs differed significantly?

Kruskal-wallis was used to compare all parks Toxocara egg data simultaneously and the results are shown in Figure 5. ANOVA was removed and this added “To compare prevalence of *Toxocara* species by zip code or borough, a spearman rank was conducted comparing the prevalence of positive parks per borough and median income per resident living in those zip codes.”
Please note that if sample sites were sampled more than once, this repeated sampling would need to be accounted for in the statistical analyses. No samples sites were repeated.
An alternative statistical approach could be Generalized Linear (Mixed) Modeling, including the predictors borough, sampling month (for reasoning see below), and sample site as a random factor (in case of repeated sampling).


Results:
The Results section needs more detail, as several aspects remain unclear. 

Did the 91 sampling sites result in 91 samples or were these sites tested multiples times? And how was the overall prevalence for NYC? In the methods it is explained that each site had 5 samples taken. More details have been added for clarity. Also added “The overall prevalence of 38.5% (35/91) contaminated playgrounds in NYC.”

It would be easier to follow if additionally the total numbers for each sampling area are given, e.g. 8/27 (29.6%) for Manhattan. This was added to Table 1.

Can the authors provide further data on the contamination intensity of the soil, like shown in Figure 5? Results of statistical analyses should also be mentioned, including which test produced which P-value. Which boroughs differed significantly – Bronx compared to all other ones?

The types of statistical analyses have been added to the methods section and the Bronx highlighted as the higher contamination intensity.

Which other parasite stages were detected besides Toxocara eggs? – there is a brief hint in the Discussion, but it is of interest which parasites were detected as they might have zoonotic potential as well. Many eggs were seen but unable to be definitively identifitied. This statement was added “Finally, other larvated parasites were seen by microscopy but unable to be definitively identified in these samples, posing a potential infectious risk to visiting animals or children.”

How was the success rate of the qPCR?

Unfortunately, the samples were not extracted properly for PCR, this was mentioned in the limitations. Only 6 of the 35 positives were also PCR positive likely due to egg loss from transfer samples from microscopy slides to PCR egg extraction.

Where the qPCR positives the samples with the highest egg counts in microscopy (please give counted egg numbers for the qPCR positive samples). 4 of 6 positive PCR had the highest levels of eggs. This is now stated in results and also the mean with range has been added.
What is the meaning of the correlation to the 40-Cycle threshold? It is a method of normalizing to a standard linear format, instead of exponential curves. Also 40-Ct has a direct relationship with egg and PCR, compared to Ct that has an inverse relationship between egg count and PCR. It is an established method of showing results. In the methods it is only mentioned that a sample was considered positive if there was detectable DNA at or before a cycle threshold of 38. Did additional samples become positive at Ct 40? Similar to our qPCR technique for parasites in stool, Toxocara DNA is extracted from eggs and qPCR to determine the dynamic range. Anything above 38 was not detected for genomic Toxocara DNA. So any sample above 38 would be consider a false positive. No samples were above 38 for cati or canis. What is the lower limit of quantification by the conducted qPCR extrapolated from the standard curve (this is an important information to draw conclusions on the possible co-existence of T. canis eggs in the qPCR positive samples or might explain failure to identify most of the samples and to detect T. canis at all)? 1 egg of either T. cani or T. cati was detected using the MP fast DNA kits for soil. This has been added to the article. Did the calculated egg count correspond to the egg count determined by microscopy? Only the slope of both curves were similar, likely due to comparing environmental sample versus eggs from cat and dog feces. This has been added to the discussion section.

It is widely described that there are seasonal variations in the occurrence of Toxocara eggs in soil. Therefore, a breakdown how the egg burden may have varied between months would be an interesting additional information (if not possible for each borough, than for the whole city). This may provide an explanation why larvated eggs could only be detected in the Bronx. As known, embryonation accelerates with ascending temperatures and humidity. So, maybe the samples in the Bronx were collected after warmer periods? This is briefly mentioned in the Discussion, but without details on when each borough was sampled it remains unclear how much this has impacted the results. Multivariate analyses (see above) would allow to tease these effects apart. Unfortunately, the samples were collected at different part of the year from different locations. Seasonal variability will be considered in future studies. These limitations have been expanded in the manuscript.

Furthermore, the correlation between median income and percent positive places could also be tested statistically.

Figure 4 legend mentions the correlation between prevalence and income, this has been added to results section also Spearman r = -0.7, p= 0.233.


Discussion:
The limitations and the bias of the conducted qPCR, which was successful in six samples only, are well and plausible discussed. In all 6 samples T. cati DNA was detected (can co-contamination with T. canis be excluded – again, what was the sensitivity of the qPCR?). Even though T. cati is described to be the predominant species contaminating playgrounds in other cities, a samples size of 6 is too low to draw a conclusion for the whole city (consider also e.g. sensitivity of the qPCR; furthermore, you cannot exclude that all other positive samples contained T. canis eggs – be more cautious with your conclusions). Agreed, the language has been made more generic. Added to discussion “Although, the samples were not optimally processed for qPCR and the lack of *T. cani* DNA maybe attributed to the small samples size.”

How can the contamination rates be classified compared to other metropoles or cities with similar climatic conditions? There are several publications available e.g. Fakhri et al., 2018: Toxocara eggs in public places worldwide - A systematic review and meta-analysis. Environmental pollution, 242:1467-1475. Thank you for the reference, it is really good paper. We have added a comparision to the Discussion section.

The human infection risk has been associated with a certain number of eggs per grams soil (e.g. Woodruff et al. 1981: Toxocara ova in soil in the Mosul District, Iraq, and their relevance to public health measures in the Middle East. Ann Trop Med Parasitol. 75:555-7). This is a relevant point when stating that infection risk was highest in the Bronx. Authors should give numbers of detected eggs in their samples in the manuscript, e.g. by including a table. Figure 5 has the amount of eggs detected by borough, this was also added to Table 1. Figure

Cleaning of play areas is mentioned - please include cleaning frequencies in public places in NYC in the MS.

Cleaning has been removed from the manuscript.

Figures/Table:
Figure 1: The provided pictures of Toxocara eggs are not well focused, please provide better pictures if possible.

These are the highest quality pictures we have.

Figure 2: As PLOS NTD is an international journal for scientists all over the world, please use the metric system. We do not have access to the ARCGIS software anymore, since the study was done in the US we have left the map using miles.

Figure 4: The income per zip code is not a series of consecutive measurements, therefore the points should not be connected via a line. 
The boroughs are connected and stratified by income, it is less confusing to have a continuous line in the Y2 axis to show continuous changes between the variables.

Figure 5: It would be nice to illustrate which boroughs differed significantly – Bronx compared to all other ones? An asterisk has been added to the Bronx in the graph and the legend.

Table 1: Please write 30.8 instead of 30.77 to be consistent with the other numbers. Are the +++ a semiquantitative measurement or just an estimation by the authors? This should be mentioned.

This has been updated and the +++ is a subjective measured by the authors.

General comments:
Write species names such as Toxocara in italics throughout (check the text, figures and references).
The manuscript should be formatted according to the journals guidelines and checked for typos.

This has been fixed.

--------------------

PLOS authors have the option to publish the peer review history of their article ([what does this mean?](https://journals.plos.org/plosntds/s/editorial-and-peer-review-process#loc-peer-review-history)). If published, this will include your full peer review and any attached files.


If you choose “no”, your identity will remain anonymous but your review may still be made public.


**Do you want your identity to be public for this peer review?** For information about this choice, including consent withdrawal, please see our [Privacy Policy](https://www.plos.org/privacy-policy).

Reviewer #1: No

Reviewer #2: No

Reviewer #3: No

__________________________________________________
In compliance with data protection regulations, you may request that we remove your personal registration details at any time. (Use the following URL: https://www.editorialmanager.com/pntd/login.asp?a=r). Please contact the publication office if you have any questions.
