# Supplementary material for: Toxocara species environmental contamination of public spaces in New York City
Source: PLoS Negl Trop Dis. 2020 May 5;14(5):e0008249. doi: 10.1371/journal.pntd.0008249 (PMC7228133; doi:10.1371/journal.pntd.0008249)
Supplement: Attachment — Submitted filename: Response to Reviewers.docx [file pntd.0008249.s002.docx]

Dear Dr Mejia,

Thank you very much for submitting your manuscript "Neglected infection of poverty: Toxocara species environmental contamination in public spaces in New York City" for consideration at PLOS Neglected Tropical Diseases. As with all papers reviewed by the journal, your manuscript was reviewed by members of the editorial board and by several independent reviewers. The reviewers appreciated the attention to an important topic. Based on the reviews, we are likely to accept this manuscript for publication, providing that you modify the manuscript according to the review recommendations.

Please prepare and submit your revised manuscript within 30 days. If you anticipate any delay, please let us know the expected resubmission date by replying to this email.

When you are ready to resubmit, please upload the following:

[1] A letter containing a detailed list of your responses to all review comments, and a description of the changes you have made in the manuscript.

Please note while forming your response, if your article is accepted, you may have the opportunity to make the peer review history publicly available. The record will include editor decision letters (with reviews) and your responses to reviewer comments. If eligible, we will contact you to opt in or out

[2] Two versions of the revised manuscript: one with either highlights or tracked changes denoting where the text has been changed; the other a clean version (uploaded as the manuscript file).

Important additional instructions are given below your reviewer comments.

Thank you again for your submission to our journal. We hope that our editorial process has been constructive so far, and we welcome your feedback at any time. Please don't hesitate to contact us if you have any questions or comments.

Sincerely,

Celia Holland

Guest Editor

PLOS Neglected Tropical Diseases

Christine Budke

Deputy Editor

PLOS Neglected Tropical Diseases

***********************

Dear Editors,

Please find our corrected manuscript per the reviewer comments.

We hope it is suitable for publication.

Rojelio Mejia

Reviewer's Responses to Questions

<b>Key Review Criteria Required for Acceptance?</b></br></br>

As you describe the new analyses required for acceptance, please consider the following:</br></br>

<b>Methods</b></br></br>

-Are the objectives of the study clearly articulated with a clear testable hypothesis stated?</br>

-Is the study design appropriate to address the stated objectives?</br>

-Is the population clearly described and appropriate for the hypothesis being tested?</br>

-Is the sample size sufficient to ensure adequate power to address the hypothesis being tested?</br>

-Were correct statistical analysis used to support conclusions?</br>

-Are there concerns about ethical or regulatory requirements being met?</br></br>

Reviewer #3: (No Response)

--------------------

<b>Results</b></br></br>

-Does the analysis presented match the analysis plan?</br>

-Are the results clearly and completely presented?</br>

-Are the figures (Tables, Images) of sufficient quality for clarity?</br></br>

Reviewer #3: (No Response)

--------------------

<b>Conclusions</b></br></br>

-Are the conclusions supported by the data presented?</br>

-Are the limitations of analysis clearly described?</br>

-Do the authors discuss how these data can be helpful to advance our understanding of the topic under study?</br>

-Is public health relevance addressed?</br></br>

Reviewer #3: (No Response)

--------------------

<b>Editorial and Data Presentation Modifications?</b></br><br/>

Use  this section for editorial suggestions as well as relatively minor modifications of existing data that would enhance clarity. If the only modifications needed are minor and/or editorial, you may wish to recommend “Minor Revision” or “Accept”.

Reviewer #3: (No Response)

--------------------

<b>Summary and General Comments</b></br></br>

Use  this section to provide overall comments, discuss strengths/weaknesses of the study, novelty, significance, general execution and scholarship. You may also include additional comments for the author, including concerns about dual publication, research ethics, or publication ethics. If requesting major revision, please articulate the new experiments that are needed.

Reviewer #3: I am comfortable with the changes made in response to my previous comments.

However, I do not agree with the title change in the revised manuscript. Leaving aside that none of the reviewers has asked for this change, human Toxocara infections are not only related to poverty, but more stringent to risk groups. For example, farmers, or veterinarians show significantly higher seropositivy than other professional groups. High prevalences in tropical countries are not only related to poverty, but also the climate, allowing eggs to become infective very fast and, additionally, enable survival in moist climates. Even though I agree that poverty (i.e. poor hygiene, but not necessarily low income in well developed countries) is one of various risk factors for humans to become infected, the study conducted by the authors is not related to the infection of poverty per se, it just shows higher egg contamination rates of sandpits in a borough with comparatively low income. If people in the Bronx are more frequently infected than in other boroughs cannot be

answered or extrapolated from contaminated sandpits only.

We have changed the title to: *Toxocara* species environmental contamination in public spaces in New York City

--------------------

PLOS authors have the option to publish the peer review history of their article (<a href="<https://urldefense.proofpoint.com/v2/url?u=https-3A__journals.plos.org_plosntds_s_editorial-2Dand-2Dpeer-2Dreview-2Dprocess-23loc-2Dpeer-2Dreview-2Dhistory&d=DwIGaQ&c=ZQs-KZ8oxEw0p81sqgiaRA&r=ZEy0IyQ0Gydtbeyd2qTjxqzEHjVUKaDuw8GJEp5nVMA&m=djPV9U6w0TxV8P-Xt1NuikeeL_7iZZjnDjArNF5dS_Q&s=xOUGR1W5Yz96BPcldyXqQ1fnBST4UhcAqPSob5CoxhE&e=> " target="_blank">what does this mean?</a>). If published, this will include your full peer review and any attached files.<br><br>

If you choose “no”, your identity will remain anonymous but your review may still be made public.<br><br>

<b>Do you want your identity to be public for this peer review?</b> For information about this choice, including consent withdrawal, please see our <a href="<https://urldefense.proofpoint.com/v2/url?u=https-3A__www.plos.org_privacy-2Dpolicy&d=DwIGaQ&c=ZQs-KZ8oxEw0p81sqgiaRA&r=ZEy0IyQ0Gydtbeyd2qTjxqzEHjVUKaDuw8GJEp5nVMA&m=djPV9U6w0TxV8P-Xt1NuikeeL_7iZZjnDjArNF5dS_Q&s=fXHVnvnuY9FMUiHEFanWlpFY3lABrpRrgVKTTMSKBLA&e=> " target="_blank">Privacy Policy</a>.

Reviewer #3: No

Figure Files:

While revising your submission, please upload your figure files to the Preflight Analysis and Conversion Engine (PACE) digital diagnostic tool, <https://urldefense.proofpoint.com/v2/url?u=https-3A__pacev2.apexcovantage.com&d=DwIGaQ&c=ZQs-KZ8oxEw0p81sqgiaRA&r=ZEy0IyQ0Gydtbeyd2qTjxqzEHjVUKaDuw8GJEp5nVMA&m=djPV9U6w0TxV8P-Xt1NuikeeL_7iZZjnDjArNF5dS_Q&s=2VIqppj5Imyx5ZNplyISGFiDIYJ1GE-xnd4Uvr8qGYg&e=> . PACE helps ensure that figures meet PLOS requirements. To use PACE, you must first register as a user. Then, login and navigate to the UPLOAD tab, where you will find detailed instructions on how to use the tool. If you encounter any issues or have any questions when using PACE, please email us at [figures@plos.org](mailto:figures@plos.org).

Data Requirements:

Please note that, as a condition of publication, PLOS' data policy requires that you make available all data used to draw the conclusions outlined in your manuscript. Data must be deposited in an appropriate repository, included within the body of the manuscript, or uploaded as supporting information. This includes all numerical values that were used to generate graphs, histograms etc.. For an example see here: <https://urldefense.proofpoint.com/v2/url?u=http-3A__www.plosbiology.org_article_info-253Adoi-252F10.1371-252Fjournal.pbio.1001908-23s5&d=DwIGaQ&c=ZQs-KZ8oxEw0p81sqgiaRA&r=ZEy0IyQ0Gydtbeyd2qTjxqzEHjVUKaDuw8GJEp5nVMA&m=djPV9U6w0TxV8P-Xt1NuikeeL_7iZZjnDjArNF5dS_Q&s=sxspfewl0N_2LBHuAx4nmzEfJyrLgoONCueDQI01BGM&e=> .

Reproducibility:

To enhance the reproducibility of your results, PLOS recommends that you deposit laboratory protocols in protocols.io, where a protocol can be assigned its own identifier (DOI) such that it can be cited independently in the future. For instructions see <https://urldefense.proofpoint.com/v2/url?u=http-3A__journals.plos.org_plosntds_s_submission-2Dguidelines-23loc-2Dmaterials-2Dand-2Dmethods&d=DwIGaQ&c=ZQs-KZ8oxEw0p81sqgiaRA&r=ZEy0IyQ0Gydtbeyd2qTjxqzEHjVUKaDuw8GJEp5nVMA&m=djPV9U6w0TxV8P-Xt1NuikeeL_7iZZjnDjArNF5dS_Q&s=uN7GCCa-BQRYDvicmCC6pj_JwTc2-k3eQuCPgYddUGs&e=>

__________________________________________________

In compliance with data protection regulations, you may request that we remove your personal registration details at any time.  (Use the following URL: <https://urldefense.proofpoint.com/v2/url?u=https-3A__www.editorialmanager.com_pntd_login.asp-3Fa-3Dr&d=DwIGaQ&c=ZQs-KZ8oxEw0p81sqgiaRA&r=ZEy0IyQ0Gydtbeyd2qTjxqzEHjVUKaDuw8GJEp5nVMA&m=djPV9U6w0TxV8P-Xt1NuikeeL_7iZZjnDjArNF5dS_Q&s=_Cse07UhjDyg66k7TPKCQgLpbgbynUQxgSEiDHChyqA&e=> ). Please contact the publication office if you have any questions.
